# Rem2-Targeted shRNAs Reduce Frequency of Miniature Excitatory Postsynaptic Currents without Altering Voltage-Gated Ca^2+^ Currents

**DOI:** 10.1371/journal.pone.0025741

**Published:** 2011-09-29

**Authors:** Hong-Gang Wang, Chuan Wang, Geoffrey S. Pitt

**Affiliations:** Division of Cardiology, Department of Medicine, and the Ion Channel Research Unit, Duke University Medical Center, Durham, North Carolina, United States of America; Sackler Medical School, Tel Aviv University, Israel

## Abstract

Ca^2+^ influx through voltage-gated Ca^2+^ channels (VGCCs) plays important roles in neuronal cell development and function. Rem2 is a member of the RGK (Rad, Rem, Rem2, Gem/Kir) subfamily of small GTPases that confers potent inhibition upon VGCCs. The physiologic roles of RGK proteins, particularly in the brain, are poorly understood. Rem2 was implicated in synaptogenesis through an RNAi screen and proposed to regulate Ca^2+^ homeostasis in neurons. To test this hypothesis and uncover physiological roles for Rem2 in the brain, we investigated the molecular mechanisms by which Rem2 knockdown affected synaptogenesis and Ca^2+^ homeostasis in cultured rat hippocampal neurons. Expression of a cocktail of shRNAs targeting rat Rem2 (rRem2) reduced the frequency of miniature excitatory postsynaptic currents (mEPSCs) measured 10 d after transfection (14 d *in vitro*), but did not affect mEPSC amplitude. VGCC current amplitude after rRem2-targeted knockdown was not different from that in control cells, however, at either 4 or 10 d post transfection. Co-expression of a human Rem2 that was insensitive to the shRNAs targeting rRem2 was unable to prevent the reduction in mEPSC frequency after rRem2-targeted knockdown. Over-expression of rRem2 resulted in 50% reduction in VGCC current, but neither the mEPSC frequency nor amplitude was affected. Taken together, the observed effects upon synaptogenesis after shRNA treatment are more likely due to mechanisms other than modulation of VGCCs and Ca^2+^ homeostasis, and may be independent of Rem2. In addition, our results reveal a surprising lack of contribution of VGCCs to synaptogenesis during early development in cultured hippocampal neurons.

## Introduction

Ca^2+^ influx through voltage-gated Ca^2+^ channels (VGCCs) controls diverse neuronal functions such as neurotransmitter release and excitation-transcription coupling. Extensive regulatory mechanisms, such as phosphorylation of the channels' pore-forming α_1_ subunits by multiple kinases [1] or interaction with calmodulin [2]–[4], fine-tune VGCC activity and the resultant Ca^2+^ influx to modulate neuronal behavior.

Recently the RGK (Rad, Rem, Rem2, Gem/Kir) subclass of small GTPases have received considerable attention as VGCC modulators because of their potent inhibitory properties, but their mechanism of action and contribution to physiology remains controversial [5]. Exogenous expression of a RGK in any cell with high voltage-activated (Ca_V_β-containing) VGCCs results in inhibition of the Ca^2+^ current in a Ca_V_β-dependent manner [6]–[9]. In addition to regulating VGCCs, Rad and Gem (but not Rem nor Rem2) can bind and inhibit Rho kinase β, and thereby affect cell-shape [10]. The RGK GTPases further stand apart from other small G proteins because they are regulated by transcription [11], [12] and only weakly, if at all, by guanine nucleotides [13]. This suggests that Ca^2+^ channel activity can be titrated through transcriptional control of endogenous RGKs [8].

While multiple studies have extensively characterized the biophysics of RGK-mediated inhibition of VGCCs and the biochemistry of interaction with Ca^2+^ channel subunits, attempts to delineate physiological roles for RGKs have been limited to three reports, only one of which focused on the brain. Knockout of Rad increased susceptibility to cardiac hypertrophy after pressure overload [14]. Whether this resulted from loss of Ca^2+^ channel inhibition was not explored, but endogenous Rad could foster tonic inhibition of cardiac Ca_V_1.2 Ca^2+^ channels as shown by shRNA knockdown in isolated myocytes [15]. Rem2, which is expressed in adult rat brain [16], was identified in an RNAi screen as a regulator of synaptogenesis [17]. RNAi or shRNA knockdown strategies targeting Rem2 in cultured neonatal hippocampal neurons decreased the density of both glutamatergic and GABAergic synapses. Although altered Ca^2+^ homeostasis after Rem2 knockdown was not assessed in that study, over-expression of Rem2 in neurons had previously been shown capable of inhibiting VGCCs [18]. Together, these results suggested a connection between calcium homeostasis, mediated by Rem2, and synapse development [17].

In an attempt to establish the physiological role of Rem2 in neurons and to address the hypothesis that RGK-mediated control of Ca^2+^ homeostasis regulates synapse development, we set out to investigate the molecular mechanisms by which Rem2 knockdown affected synaptogenesis. The reproducibility of synaptogenesis readouts and the ability to measure VGCC currents in cultured hippocampal neurons offered an opportunity to explore the roles of endogenous RGKs and their effects upon VGCCs and Ca^2+^ homeostasis in excitable cells.

## Materials and Methods

### Ethics Statement

Animals were handled according to National Institutes of Health Guide for the Care and Use of Laboratory Animals and approved by Duke University Animal Care and Welfare Committee (approval A315-10-2).

### Molecular Biology

The GFP-rRem2 and GFP-hRem2 were constructed by subcloning rat Rem2 (rRem2) or human Rem2 (hRem2) into the first multiple cloning site (MCS) in pEGFP-C1 (Clontech, CA). Rat Rem2 shRNAs cloned into the pSuper shRNA expression vector (OligoEngine, WA) were kindly provided by M.E. Greenberg (Harvard) [17].

### Neuronal Cultures and Electrophysiology

Hippocampi from 1–2 d newborn rat (Sprague Dawley strain) were dissociated and plated on glass coverslips in 12-well cell culture plate in the density of 100,000/ml as described previously [19]. The cells in the wells were randomly assigned into experimental groups. Hippocampal neurons 4 d *in vitro* (DIV) were transfected with either GFP or GFP-rRem2 for the experiments assessing Rem2 overexpression; or co-transfected with GFP and pool of the three shRNA constructs targeting rRem2 using calcium phosphate precipitation as described [17]. The pSuper plasmid without an insert was used as a control. In the rescue experiments, GFP-hRem2 and shRNAs were co-transfected. Electrophysiological recordings were performed at 3, 4, and 10 d after transfection (7, 8, 14 DIV). mEPSCs and VGCC currents were obtained at room temperature from GFP-positive cells in the whole-cell voltage patch-clamp configuration with an Axopatch 200B amplifier. For mEPSC recordings the pipette internal solution contained the following (in mM): 120 potassium gluconate, 10 KCl, 5 MgCl_2_, 0.6 EGTA, 5 HEPES, 0.006 CaCl_2_, 10 phosphocreatine disodium, 2 Mg-ATP, 0.2 GTP, and 50 U/ml creatine phosphokinase, pH 7.2; the external solution contained (in mM): 119 NaCl, 3 KCl, 20 HEPES, 2 CaCl_2_, 2 MgCl_2_, 30 glucose, 0.001 tetrodotoxin, 0.001 glycine and 0.1 pictrotoxin, pH 7.3. For VGCC current recordings, the pipette solution contained (in mM): 135 CsMeSO_3_, 5 CsCl, 5 EGTA, 1 MgCl_2_, 4 Mg-ATP, and 10 HEPES, pH 7.3; recording solution contained (in mM): 115 NaCl, 3 KCl, 10 HEPES, 5 CaCl_2_, 2 MgCl_2_, 10 glucose, 0.0005 tetrodotoxin, 20 tetraethylammonium chloride, and 5 4-aminopyridine, pH 7.3. Currents were sampled at 10 kHz and filtered at 2 kHz. VGCC currents were induced by a ramp step from −80 mV to +50 mV over 500 ms after a 50 ms step to −80 mV from a holding potential of −70 mV. Series resistance ranged from 6–20 MΩ without compensation.

### PCR Quantitative Analysis

Total mRNA was purified from neurons cultured for the indicated times using RNAeasy Plus Mini kit (Qiagen, CA). Real-time PCR quantification was performed using the BIO-RAD iCycler system (Bio-Rad Laboratories, CA). At the completion of PCR (a total of 45 cycles), the relative amount of target message in each reaction was determined from the detection threshold cycle number (Ct), which is inversely correlated with the abundance of the message's initial level, which was normalized to the Ct for actin, obtained simultaneously.

### GFP Immunoblot

HEK293 cells were washed with ice-cold TBS (150 mM NaCl and 50 mM Tris, pH 7.5), harvested, and resuspended in ice-cold TBS with 1% Triton X-100 and protease inhibitor 2 d after the cells had been transfected with the indicated combination of plasmids. Cells were lysed by pipetting up and down and then centrifuged at 17,000 X g for 10 min. Protein was then separated by SDS-PAGE, transferred to nitrocellulose, and immunoblotted with anti-GFP antibody (Covance, Princeton, NJ) and detected by chemiluminescence with SuperSignal West Pico (Pierce) on a Kodak Image Station 4000R Pro (Carestream Health, NY). Quantification of protein was performed by measuring intensity of the bands using KODAK MI software.

### Immunocytochemistry

Hippocampal neurons plated on coverslips at 4 DIV were transfected with either GFP or GFP-rRem2; or co-transfected with GFP and pool of the three shRNAs targeting rRem2 or GFP and pSuper plasmid without an insert as a control. The neurons were fixed with 4% paraformaldehyde in phosphate buffer saline (PBS) 10 d after transfection (14 DIV) and permeabilized with 0.02% saponin in PBS. After blocking with 10% bovine serum albumin (BSA) in PBS the fixed neurons were incubated with 1∶100 goat polyclonal antibody against Rem2 (C-13; Santa Cruz Biotechnology, CA) in PBS with 5% BSA over night at 4°C. Secondary Cy3-conjugated bovine anti-goat IgG antibody (Jackson ImmunoResearch Laboratory, PA) was applied at 1∶1,000 at room temperature for 1 h. Coverslips were mounted on slides with fluoromount-G (SouthernBiotech, AL). Images were obtained on a Zeiss LSM 510 inverted confocal microscope with a 40x/1.30 oil objective (Duke Light Microscopy Core Facility) by sequential scanning excited with 488 nm Argon and 561 nm Diode lasers and filtered at the range of 505–550 nm for GFP and 575 nm for Cy3. A 118 µm pinhole was used for both green and red channels. Focus was adjusted using the GFP signal. Laser intensity and offset for Cy3 red channel were optimized based on preliminary images from neurons transfected with GFP-Rem2 (over-expression). A laser intensity of 5.9% with detector gain 601 and offset -0.472 was set for red channel for all images acquired. By using this setting we ensured that Cy3 fluorescent signal for all images obtained from different neurons was not saturated. Four scans were digitally averaged. Images were analyzed with ImageJ 1.35p (NIH). The mean fluorescent intensity within the soma was corrected by subtracting a background value, which was calculated as the mean+2SD of 4 random local areas outside of the soma.

### Statistics

All data analyses were performed using Microsoft Excel 2007. Numerical averages are presented as mean ± SEM. Unless otherwise stated, statistical significance was calculated using the unpaired two-sample Student's *t* test.

## Results

**Figure 1:**
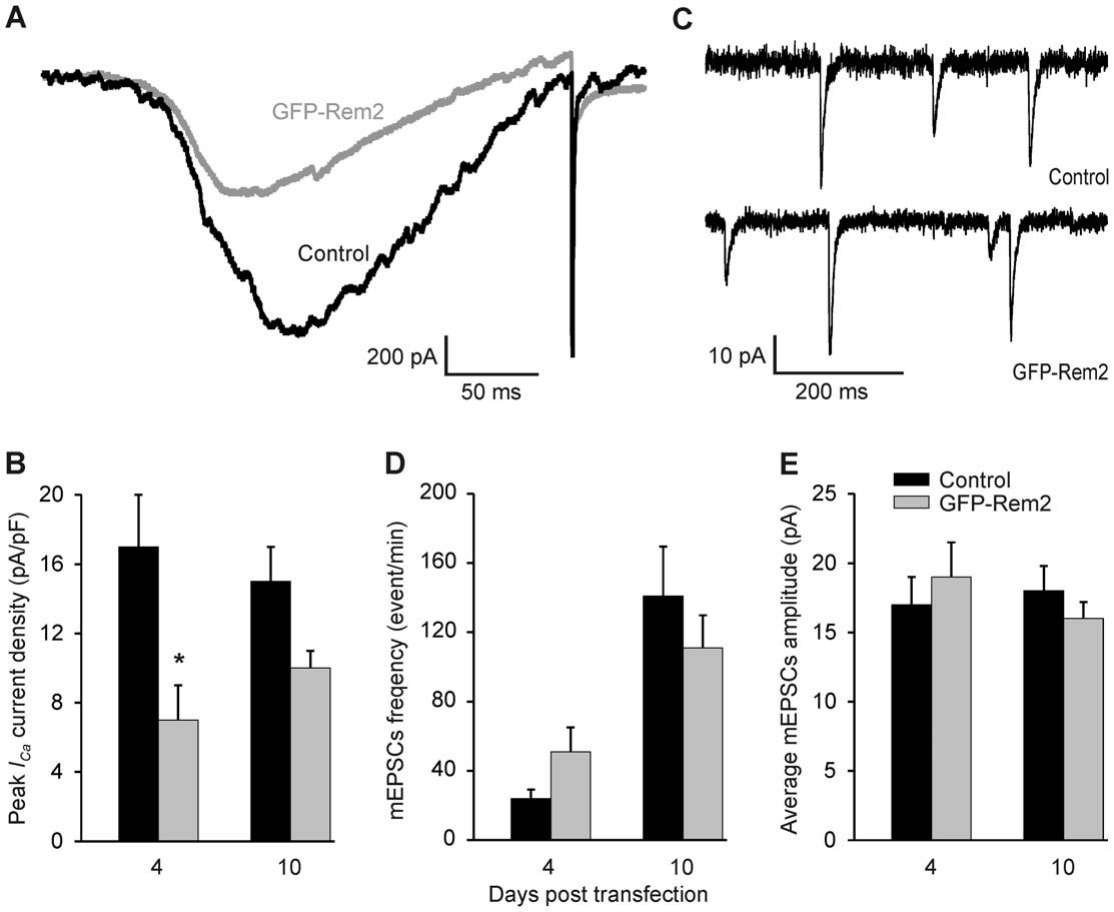
Over-expression of Rem2 reduced VGCCs without changes in mEPSCs in cultured hippocampal neurons. *A,* Exemplar VGCCs were recorded from neurons transfected with GFP-rRem2 or GFP. The currents were induced by a ramp protocol −80 mV to +50 mV over 500 ms after a 50 ms step to −80 mV from a holding potential of −70 mV. *B,* Summarized VGCC currents recorded 4 (N = 7) and 10 d (N = 8) after Rem2 over-expression, showing VGCCs decreased markedly at 4 d (p = 0.03) and there was not significant difference at 10 d (p = 0.1). *C,* Exemplar recording of mEPSCs from neurons transfected with GFP-rRem2 or GFP only. *D,* and *E*, mEPSCs showed no change in mEPSCs frequency or amplitude at 4 d post-transfection (N = 9–10; frequency p = 0.11; amplitude p = 0.53) or 10 d post-transfection (N = 15; frequency p = 0.19; amplitude p = 0.36).

To determine whether the effects upon synaptogenesis by Rem2 knockdown could be mediated by alterations in calcium homeostasis, we first tested whether rRem2 was capable of inhibiting VGCCs in cultured rat hippocampal neurons. As expected from previous reports using sympathetic and dorsal root ganglion neurons and other cell types [8], [18], over-expression of rRem2 decreased VGCC currents in hippocampal neurons. We observed more than 50% reduction of VGCC currents 4 d after transfection (Fig. 1, *A* and *B*). Current amplitude was still reduced 6 d after transfection (not shown), but reduction 10 d after transfection was no longer significant (Fig. 1*B*). Surprisingly, neither the miniature excitatory postsynaptic current (mEPSC) frequency nor amplitude was affected by Rem2 over-expression in hippocampal neurons (Fig. 1, *C*, *D*, and *E*), despite the marked reduction in VGCC currents. Thus, the signaling pathways mediating synaptogenesis are tolerant of a significant reduction in whole-cell VGCC activity.

**Figure 2:**
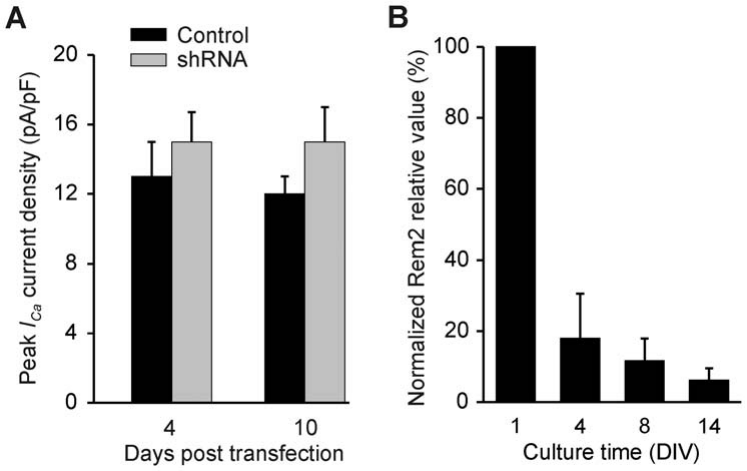
Rat Rem2 shRNAs did not change VGCC peak currents in hippocampal neurons. *A,* VGCC currents from control neurons and neurons transfected with rRem2-targeted shRNAs. There was no change in current density at either 4 d post-transfection (N = 11, p = 0.24) or 10 d post-transfection (N = 5–7, p = 0.48). *B,* Rem2 mRNA relative values in hippocampal neuron culture was measured at 1, 4, 8 and 14 DIV (N = 5–9) with PCR quantitative analysis. The relative value was normalized to the value of 1 DIV.

Since rRem2 was able to reduce VGCC currents, we tested whether rRem2 shRNA relieved tonic inhibition of VGCCs by endogenous Rem2. We measured whole-cell VGCC currents in cultured rat hippocampal neurons at 4 and 10 d after expression of a cocktail of shRNAs targeting rRem2. Current amplitude from neurons with expression of the control plasmid (empty vector) was not different from non-transfected cells (14.6±1.0 pA/pF vs. 13.3±1.6 pA/pF, P = 0.51), showing that the transfection procedure had no effect on the VGCC. Nor, however, was current amplitude after rRem2-targeted knockdown in neurons different from that in control neurons transfected with empty vector at either 4 or 10 d post transfection (Fig. 2*A*). This was not because the shRNA was incapable of reducing Rem2 protein (see data below showing that shRNA was effective at reducing Rem2 protein expressed in HEK cells; an equivalent immunoblot analysis of Rem2 protein after knockdown in culture hippocampal neurons was not possible because of the comparatively low transfection efficiency in neurons, but see immunostaining analysis below).

A possible explanation for the lack of efficacy of Rem2 shRNA was that the level of Rem2 mRNA in these cultures was negligible; thus, even effective targeting of Rem2 would have little effect upon Rem2 protein levels and thus upon VGCC currents. To investigate this possibility, we first performed semi-quantitative PCR to measure the relative amounts of Rem2 mRNA in cultured hippocampal neurons at 1, 4, 8 and 14 DIV (Fig. 2*B*). We found that the amount of Rem2 mRNA decreased by >80% by day 4 *in vitro* (when transfection with shRNA was performed for the physiology experiments) and by 14 DIV was further reduced to <10% of the level at 1 DIV. Thus, there was comparatively little Rem2 mRNA to target with shRNA at the critical time period in which transfection was performed.

**Figure 3:**
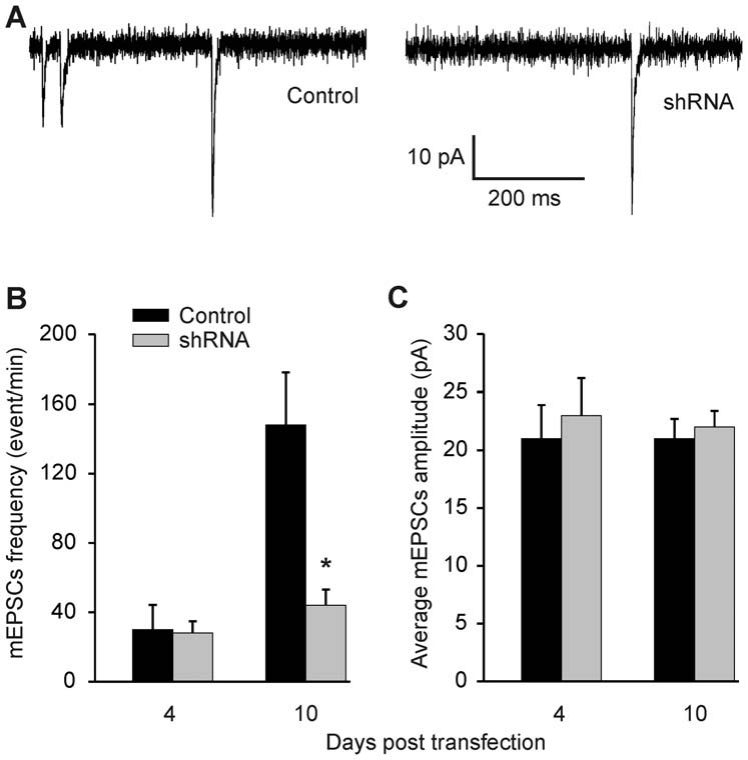
Rat Rem2 shRNAs reduced mEPSCs frequency in hippocampal neurons. *A,* Exemplar recording of mEPSCs from neurons transfected with rRem2-targeted shRNAs or the control plasmid. *B,* and *C,* Summarized mEPSCs frequency and amplitude 4 (N = 6) and 10 d (N = 12) after transfection, showing that the mEPSCs frequency was reduced significantly at 10 d after transfection of shRNAs compared with control transfection (p = 0.006). There was no change in amplitude (p = 0.55).

So, how does knockdown of Rem2 affect synaptogenesis? To attempt to address this question, we first confirmed that transfection of a cocktail of shRNAs targeting rRem2 in our cultured neurons reduced the frequency of spontaneously occurring mEPSCs, as previously reported [17]. Indeed, the shRNA cocktail effectively reduced mEPSC frequency measured 10 d after transfection (14 DIV), as shown in Fig. 3*A*–*B*. The reduction in mEPSC frequency took time to manifest; no difference in frequency was detected 4 d after shRNA transfection (Fig. *3B*). ShRNA did not affect mEPSC amplitude (Fig. *3C*). Expression of the control plasmid (empty vector) had no effect on mEPSC frequency when compared to non-transfected cells (148±30 event/min vs. 103±18 event/min, P = 0.22). Since we did not observe a correlation between shRNA-mediated reduction in mEPSC frequency and relief of tonic VGCC inhibition (Fig. 2*A*), the observed reduction in mEPSC frequency after shRNA treatment was more likely due to mechanisms other than modulation of VGCCs and Ca^2+^ homeostasis.

**Figure 4:**
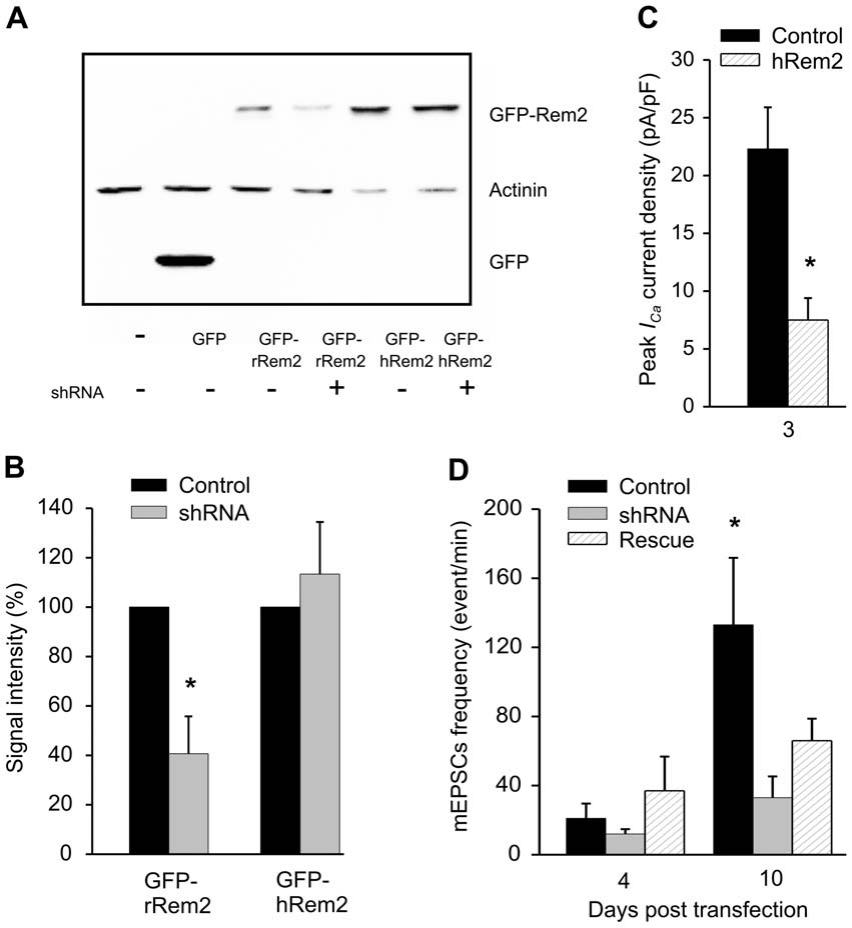
GFP-hRem2, which was insensitive to rRem2-targeted shRNAs, could not rescue the reduction in mEPSCs frequency induced by shRNAs. *A,* and *B*, immunoblot for GFP of lysates of untransfected HEK cells or cells transfected with GFP, GFP-rRem2 plus either rRem2 shRNAs or empty vector, and GFP-hRem2 plus either rRem2 shRNAs or empty vector. The rRem2-targeted shRNAs reduced GFP-rRem2 expression (N = 4, p = 0.03). *C,* VGCC currents recorded from cultured hippocampal neurons 3 d after transfection with GFP-hRem2 (N = 8) or GFP (N = 7). GFP-hRem2 reduced VGCC currents (p = 0.03). *D,* mEPSC frequency recorded at 4 d (N = 8) and 10 d (N = 16–21) after transfection with empty vector, rRem2-targeted shRNAs, or rRem2-targeted shRNAs together with GFP-hRem2. Transfection with rRem2-targeted shRNAs reduced mEPSCs frequency at 10 d compared to control (p = 0.02). Co-transfection with GFP-hRem2 did not rescue the effect of rRem2-targeted shRNAs (p = 0.12).

An alternative scenario is that the effects of the rRem2-targeting shRNAs were independent of Rem2. To test for this possibility, we attempted a rescue experiment in which we expressed a shRNA-insensitive hRem2 (amino acid sequence is 93% identical) along with the rRem2-targeted shRNAs. We first confirmed that the shRNAs were capable of reducing levels of rRem2 protein while sparing hRem2. In HEK cells expressing a GFP-tagged rRem2, the rRem2-targeted shRNAs reduced protein 59% (compared to control); protein levels of a GFP-tagged hRem2 were unaffected (Fig. 4, *A* and *B*). We then confirmed that the GFP-hRem2 was active in rat hippocampal neurons: when over-expressed, it reduced whole-cell VGCC currents by 66% (Fig. *4C*). Although GFP-hRem2 was capable of reducing VGCC currents and was insensitive to the shRNAs targeting rRem2, it was unable to prevent the reduction in mEPSC frequency by the rRem2-targeted shRNAs, however. As shown in Fig. 4*D*, and consistent with data in Fig. 3 and a previous report [17], the rRem2-targeting shRNAs reduced the mEPSC frequency in hippocampal neurons 10 d post transfection compared to control (vector only) neurons, but co-expression of GFP-hRem2 failed to restore the mEPSC frequency to control cell levels.

**Figure 5:**
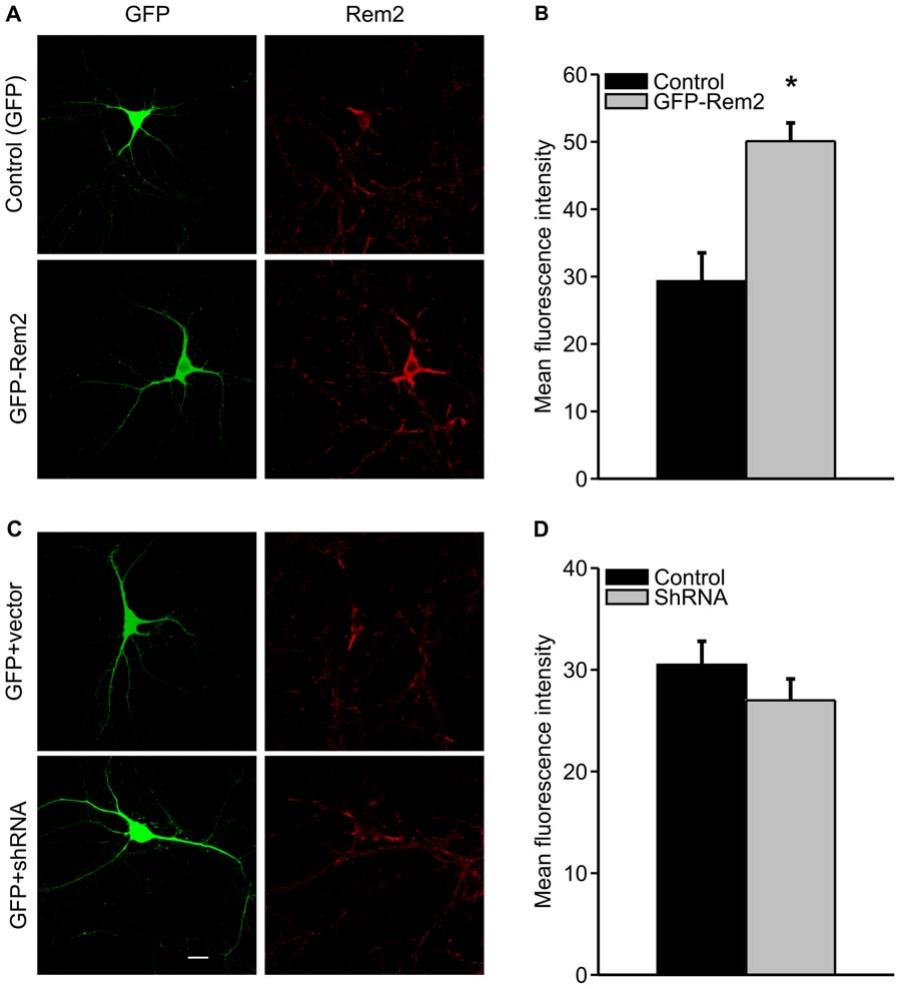
shRNA did not reduce endogenous Rem2 measured by immunocytochemistry in hippocampal neurons. *A* and *C,* Exemplar images of neurons transfected with GFP or GFP-rRem2; or co-transfected with GFP and control vector *versus* GFP and a pool of rRem2 shRNAs. Left column, expressed GFP; right column, rRem2 detected with antibody against Rem2 and visualized with secondary antibody Cy3. Scale bar, 20 µm. *B* and *D,* Summarized GFP and Cy3 fluorescent levels indicated with mean gray value. Neurons overexpressing rRem2 (n = 41) showed significantly stronger fluorescent signal than control neurons (N = 15) (p = 0.0003). There was not significantly difference between neurons transfected with rRem2 shRNA (N = 51) and control plasmid (N = 48) (p = 0.25).

This suggested the possibility that shRNA, although reducing mEPSC frequency, did not affect endogenous Rem2 protein. We tested this hypothesis by quantifying Rem2 immunostaining in hippocampal neurons after shRNA treatment with an antibody that can detect a ∼ 35 kDa protein (the predicted mass of Rem2) in mouse brain tissue extracts (see manufacturer's data). First, we tested whether the antibody was also capable of detecting Rem2 in cultured hippocampal neurons by comparing immunofluorescence within GFP-positive neurons transfected with GFP-tagged rRem2, which we showed was functional as evidenced by its ability to reduce Ca^2+^ current in transfected neurons (see Fig. 1). In the soma of neurons transfected with GFP-rRem2 we observed significantly higher immunofluorescence compared to control neurons transfected with GFP only (Fig. 5*A*–*B*). No difference in the pattern of immunofluorescence was observed in the dendritic arbor. We then tested whether shRNA diminished endogenous somatic Rem2 under the same conditions that reduced mEPSC frequency and observed no difference in the level of immunofluorescence (Fig. 5*C*–*D*) when compared to control treated cells. Together, the inability of the GFP-hRem2 to restore the mEPSC frequency and the lack of a detectable effect of shRNA on endogenous Rem2 levels suggest that the effects of the shRNAs upon mEPSCs are independent of Rem2.

## Discussion

The physiologic roles of RGK proteins remain poorly understood despite recent studies elucidating the molecular mechanisms by which RGKs inhibit VGCCs [9]. Previous explorations of RGK roles in physiology have relied almost exclusively upon over-expression in cell culture systems or in transgenic animals [20] and have not addressed the roles of endogenous RGKs. Exceptions are the set of experiments demonstrating that knockout of Rad increased susceptibility to cardiac hypertrophy [14] and, separately, that knockdown of Rad in cardiac myocytes increased L-type VGCCs [15]. At least in heart, those data together suggest that Rad is capable of tonic inhibition of VGCCs and that excessive relief of that inhibition (by Rad knockout) is pathologic. How Rad is regulated under physiologic conditions in heart, and the consequent effects upon VGCCs has not been examined, but remains an exciting area for future study.

In this context, the reported role for Rem2 on synaptogenesis [17] offered a new insight into RGK physiology and was a major motivation for this study. Rem2 knockdown in cultured hippocampal neurons decreased mEPSC frequency—effect that was replicated in this study. The lower mEPSC frequency suggests either a reduction in release probability in the presynaptic neuron or a decreased number of synaptic contacts on the postsynaptic neuron in which recordings were performed [21]. Since transfection efficiency was relatively low (∼20 – 30%), it is unlikely that the observed effects were presynaptic; thus, we assume that the effects were limited to a decrease in the number of synapses generated on the postsynaptic neuron in which mEPSCs were recorded. How Rem2 could affect synapse formation in a postsynaptic neuron is unclear, but several lines of evidence from our results suggest that the mechanism does not include regulation of Ca^2+^ homeostasis through inhibition of VGCCs. First, overexpression of Rem2 and a resultant decrease in VGCC currents failed to affect either mEPSC frequency or amplitude. Second, expression of shRNAs targeting Rem2 did not affect VGCC currents. Rather, our results suggest that the reduction in mEPSCs after shRNAs targeting Rem2 may be independent of Rem2, since the shRNAs also reduced mEPSC frequency in the presence of a shRNA-insensitive Rem2 and we could not detect any change in the level of Rem2 after shRNA under same conditions that produced changes in synaptogenesis. While it is possible that the hRem2 failed to complement the reduction in rRem2 in rat hippocampal neurons, the 93% identity between the 2 proteins suggests that this was an unlikely scenario. Moreover, hRem2 was equally effective as rRem2 in reducing VGCC current after over-expression, demonstrating that hRem2 was functional in hippocampal neurons, at least for this well-established measure of Rem2 efficacy. The failure of the rescue construct in these experiments is significant since it has been demonstrated shRNAs can affect synapses through activation of innate antiviral response pathways—independent of the shRNA target—leading specifically to loss of dendritic spines and a reduction in mEPSCs [22]. In contrast to our failed attempts, it was recently shown that the reduction in synapses after Rem2 shRNA knockdown, as measured by immunocytochemical techniques, could be rescued by a shRNA-resistant Rem2 [23]. The reasons for the different results are not clear, but one possibility may be distinctions in the way synaptogenesis was quantified. We measured a functional readout, mEPSCs, while the other report measured parameters such as dendritic density. As noted above, the failure to rescue in our experiments cannot be attributed to lack of efficacy of the hRem2 rescue construct, since we were able to confirm that hRem2 was functional as a VGCC inhibitor. Thus, the discrepancy between the rescue results could imply that morphological changes (measured by Ghiretti and Paradis [23]) and the functional changes measured in this report are independent. Another difference between these reports was that we were unable to detect an effect of shRNA on endogenous Rem2 immunostaining, using the same shRNA constructs and under the same conditions that produced the functional readout (a reduction in mEPSCs) by which Rem2 was originally identified as a mediator of synaptogenesis [17]. This discrepancy may derive from the different primary antibodies used (in this report, a commercial antibody demonstrated to detect a protein of the predicted molecular weight within adult mouse brain lysates; Ghiretti and Paradis used a custom antibody [23]). Thus, the failure of the shRNA to affect VGCCs in our experiments, no detectable change in Rem2 by immunostaining after shRNA, and the lack of any effect on mEPSCs observed after Rem2 over-expression provides little support for a Rem2-mediated effect. We considered the possibility that the Rem2 shRNAs targeted other mRNAs, but search of available databases did not identify any other targets (not shown).

Rem2 is expressed in human embryonic stem cells, in which it maintains the cell cycle and controls proper differentiation towards ectoderm, suggesting a role in neuronal development [24] but its expression pattern during neonatal development in the brain is not known. We tested Rem2 mRNA levels with quantitative RT-PCR in hippocampal neuron cultures and found that the relative level of Rem2 mRNA declined by ∼80% at 4 DIV compared to 1 DIV, and that the level remained low level through 14 DIV (the extent of our experimental time course). The period between 4 DIV and 12 DIV is a critical period for the formation of functional synapses, as indicated by data that only 11% of neuron pairs have evoked synaptic transmission at 4 DIV compared to 75% by 12 DIV [25]. While we cannot be certain that the low level of Rem2 mRNA correlates to a low protein level, those data do suggest that there is not a significant transcriptional regulation of Rem2 during this important developmental time.

Another interesting outcome of our experiments is that the 50% reduction in VGCC activity for up to 6 days in culture (after Rem2 overexpression) did not alter synaptogenesis. That development of synapses can tolerate a significant and sustained reduction in Ca^2+^ influx through VGCCs suggests two possibilities: either that any role for elevated intracellular Ca^2+^ in synaptogenesis is supported through other Ca^2+^ entry sources or that Rem2 overexpression triggered compensatory increases through other entry sources. In support of the former hypothesis is extensive evidence that VGCCs, especially Ca_V_1.2 L-type Ca^2+^ channels, have prominent roles in activity-dependent gene expression, but other Ca^2+^ entry sources appear to play more prominent roles during early development [26].

In conclusion, our results showed that Rem2 did not exhibit any observable tonic inhibitory modulation of VGCCs in cultured hippocampal neurons and that inhibition of VGCCs during early development did not affect synaptogenesis. Roles for Rem2 in other aspects of neuromodulation remain unexplored, and whether these roles are mediated through regulation of VGCC activity will be an interesting area of future study.
